# Increased Growth Factors Play a Role in Wound Healing Promoted by Noninvasive Oxygen-Ozone Therapy in Diabetic Patients with Foot Ulcers

**DOI:** 10.1155/2014/273475

**Published:** 2014-06-24

**Authors:** Jing Zhang, Meiping Guan, Cuihua Xie, Xiangrong Luo, Qian Zhang, Yaoming Xue

**Affiliations:** Department of Endocrinology & Metabolism, Nanfang Hospital, Southern Medical University, Guangzhou, Guangdong 510515, China

## Abstract

Management of diabetic foot ulcers (DFUs) is a great challenge for clinicians. Although the oxygen-ozone treatment improves the diabetic outcome, there are few clinical trials to verify the efficacy and illuminate the underlying mechanisms of oxygen-ozone treatment on DFUs. In the present study, a total of 50 type 2 diabetic patients complicated with DFUs, Wagner stage 2~4, were randomized into control group treated by standard therapy only and ozone group treated by standard therapy plus oxygen-ozone treatment. The therapeutic effects were graded into 4 levels from grade 0 (no change) to grade 3 (wound healing). The wound sizes were measured at baseline and day 20, respectively. Tissue biopsies were performed at baseline and day 11. The expressions of vascular endothelial growth factor (VEGF), transforming growth factor-*β* (TGF-*β*), and platelet-derived growth factor (PDGF) proteins in the pathologic specimens were determined by immunohistochemical examinations. The effective rate of ozone group was significantly higher than that of control group (92% versus 64%, *P* < 0.05). The wound size reduction was significantly more in ozone group than in control group (*P* < 0.001). After treatment, the expressions of VEGF, TGF-*β*, and PDGF proteins at day 11 were significantly higher in ozone group than in control group. Ozone therapy promotes the wound healing of DFUs via potential induction of VEGF, TGF-*β*, and PDGF at early stage of the treatment. (Clinical trial registry number is ChiCTR-TRC-14004415).

## 1. Introduction

Diabetic foot ulcer (DFU) has been increasingly recognized as one of the major complications of diabetes mellitus (DM), which is associated with a very high mortality and morbidity [1]. The management of DFUs has been an open problem for a long time in the world. Most recently a retrospective cohort study, with 312,744 wounds of all types in 154,664 patients enrolled, shows that most wounds were DFUs (19.0%), venous leg ulcers (26.1%), and pressure ulcers (16.2%) [2]. Considering the high costs associated with treating DFUs, the development of better treatment strategies is warranted.

The medical use of ozone (also known as triatomic oxygen and trioxygen) was initiated in the 19th century. Ozone has multiple therapeutic effects in wound healing due to the property of releasing nascent oxygen, which has been shown to have bactericidal capabilities and to stimulate antioxidant enzymes [3–6]. Although the ozone treatment improves the diabetic outcome [7], there are few prospective randomized clinical trials to verify the efficacy of the noninvasive oxygen-ozone treatment on the wound healing at the early stage of treatment in DFUs. According to the results of the largest DFUs clinical trial, the DFUs median time to heal for weekly or higher-frequency debridement was 21 days [4]. At the early stage of treatments (including debridement), wound healing is managed by platelet-derived growth factors (PDGFs) and transforming growth factors-*β* (TGF-*β*). And then tissue macrophages supply the key multiple growth factors for healing [8, 9]. However, limited research has been conducted on the early efficacy of treatments as early as day 20 after treatment in DFUs. Also it is difficult to obtain the tissue specimens in clinical trials to verify the correlation of the treatments and the expressions of growth factors in the local wounds.

In the present study, we conducted a prospective randomized controlled clinical study in diabetic patients complicated with DFUs to assess the effects of ozone therapy on the healing and the expressions of VEGF, TGF-*β*, and PDGF of the wounds at the early stage after treatment.

## 2. Methods

### 2.1. Ethics Statement

This study was approved by the ethics committee of Nanfang Hospital, Southern Medical University. All patients gave signed informed consent prior to participating in the study.

### 2.2. Study Participants

Hospitalized patients with type 2 diabetes mellitus (T2DM) were recruited from the Department of Endocrinology and Metabolism, Nanfang Hospital, Southern Medical University, during March 2012 to January 2013. A total of 50 patients, aged 18 yrs or older, with DFU of Wagner classification stages 2, 3, or 4 were included in the study. Patients were excluded from the study if they had one or more of the following conditions [10]: (1) gangrenous ulcers in whole foot, (2) active osteomyelitis, (3) a history of collagen diseases, (4) hyperthyroidism, (5) pregnancy or nursing, (6) hemoglobin A1c (HbA1c) levels >10.5%, (7) ankle brachial index (ABI) <0.70, (8) hemoglobin less than 90 g/L, (9) liver function tests (alanine transaminase, aspartate transaminase, or c-glutamyl transpeptidase) elevated to more than three times the upper normal limit, (10) serum creatinine >133 *μ*mol/L or dialysis, and (11) a known allergy to ozone. Patient demographics and clinical characteristics were obtained from medical records including age, gender, duration of DM, duration of DFU, systolic blood pressure (SBP)/diastolic blood pressure (DBP), HbA1c, hemoglobin (Hb), glutamic pyruvic transaminase (ALT), creatinine (Cr), and ABI.

### 2.3. Study Design

Included patients were randomized into two groups. After debridement, the ozone group received noninvasive oxygen-ozone treatments with 52 *μ*g/mL ozone (total volume: 20–50 mL) in a special bag for 30 min per day for 20 days using the ozone generator device (Humazon Promedic, German) in addition to standard treatment. The control group received only standard treatment which included debridement once every two days and wound dressings appropriate for the degree of exudate and moisture maintenance of the wound. A special technician was trained to operate the ozone generator device and performed the actual treatments. Study visits were performed at baseline and at days 11 and 20. At each visit, photography of the ulcers was taken at a distance of 20–30 cm and in the same light; then the wound condition, length, width, depth, healing progression, presence of infection, and the need for debridement were assessed. Ulcer areas were calculated from film transparency tracings using grid paper.

### 2.4. Criterion of Therapeutical Effect

**Figure 1:**
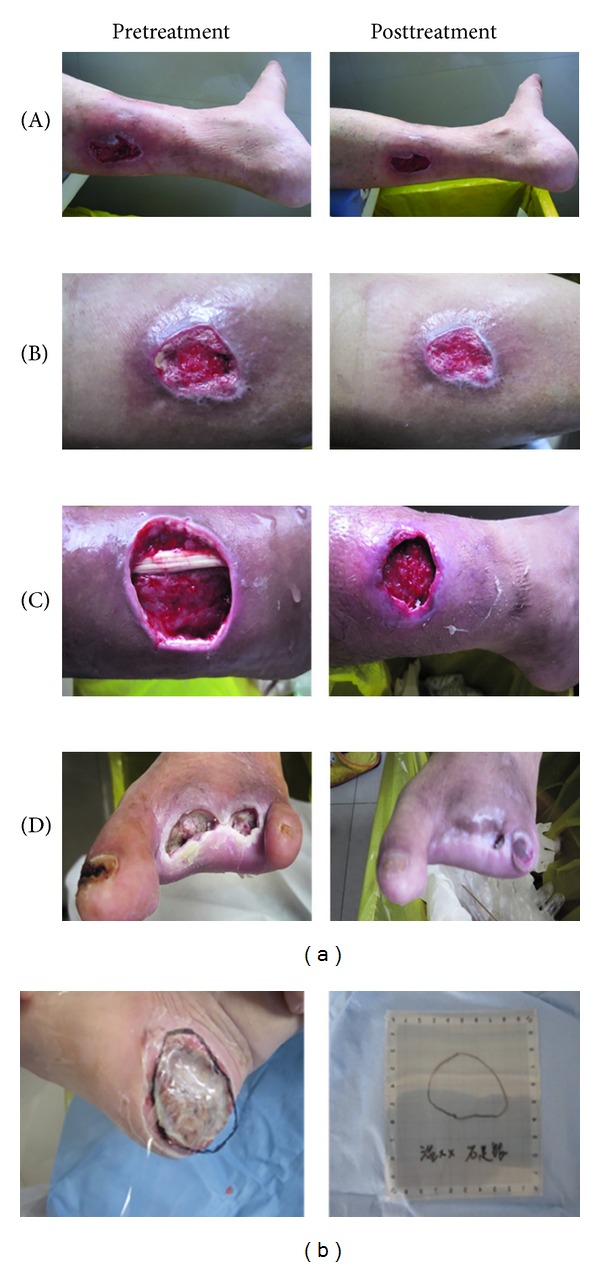
Criteria of therapeutic effects and wound size determination. Panel (a) (A) Grade 0: no change or worse than before. (B) Grade 1: wound size reducing less than 1/2. (C) Grade 2: wound size reducing more than 1/2, the secretion obviously less than before, there is little necrosis, fresh granulation generated. (D) Grade 3: wound healing, completely epithelialized. Panel (b) Ulcer areas were calculated from film transparency tracings using grid paper.

At each visit the wound condition was evaluated into one of the four grades by the criteria as follows (Figure 1). Grade 0: no change or worse than before; Grade 1: wound size reducing less than 1/2; Grade 2: wound size reducing more than 1/2, the secretion obviously less than before, there is little necrosis, fresh granulation generated; Grade 3: wound healing, completely epithelialized with dimensions at 0 × 0 × 0 cm.

### 2.5. Tissue Biopsies and Pathologic Examinations

Tissue specimens were obtained from the border area of foot ulcers, with dimensions approximately 5 mm × 5 mm × 1 mm (length × width × depth), comprising the ulcer edge and surrounding skin at day 0 and day 11. Half of the tissue was weighed and homogenized in 3 mL PBS (pH 7.4) followed by centrifugation. The supernatants were collected and stored at −80°C freezer for the determination of VEGF, TGF-*β*, and PDGF by ELISA kit (R&D Systems). Each specimen was applied in duplicate. The levels of VEGF, TGF-*β*, and PDGF were expressed in pg/mg. Values were expressed as mean ± SD, whereas the other half was fixed in 4% phosphate-buffered formalin and paraffin embedded. The paraffin-embedded specimens were cut into 4 mm sections and placed on silane-coated glass slides. Hematoxylin & eosin (H&E) and Masson's staining were, respectively, performed in all patients' specimens to detect the collagen deposition.

### 2.6. VEGF, TGF-*β*, and PDGF Levels in Wound Exudates by ELISA Assay

The wound exudates about 0.5 mL were collected by 1 mL-syringes at days 3, 7, and 11 after treatment in both groups. The samples were added into incubating tubes. The concentrations of VEGF, TGFF-*β*, and PDGF of supernatants were measured by using ELISA kit (R&D, USA).

### 2.7. Expressions of VEGF, TGF-*β*, and PDGF Proteins by Immunohistochemical Examinations

Tissues from each patient were fixed in formalin and embedded in paraffin. After the paraffin sections were deparaffinized, they were heated for 20 min at 105°C in antigen retrieval buffer (Jinqiao Zhongshan BioTech, Beijing, China). After blocking with 10% goat serum, the slides were incubated with each primary polyclonal antibody against VEGF (Abcam, UK), PDGF (Santa cruz, USA), and TGF-*β* (Abcam, UK), respectively, overnight at 4°C followed by horseradish peroxidase-labeled secondary antibodies for 1 h at room temperature. And then, the slides were developed with diaminobenzidine tetrahydrochloride (DAB) and counterstained with hematoxylin. For each slide, at least 5 fields were analyzed with high power (×400 magnification) microscopy by two pathologists. Specimens were defined as positive if there were cells distinctly stained by the antibodies. Immunolabeling was assessed using an Olympus CKX41 light microscope (Tokyo, Japan) and photographed using the image Pro plus win32.

### 2.8. Statistical Analysis

Statistical analyses were carried out using SPSS v.16.0. Data are shown as mean ± SD. The *t*-test for independent samples was used for a given variable's distribution to compare continuous data by treatment group. Paired *t*-test was used to compare the data before and after treatment in the same group. Proportions were compared using Chi-square test. Significance was set at *P* < 0.05.

## 3. Results

### 3.1. Patient Dispensation and General Data at Baseline

As indicated in Figure 3, 50 patients with DFU were randomized to ozone group (*n* = 25) and control group (*n* = 25). All of these patients completed the study visits. There were no significant differences between two groups in patient demographics and clinical characteristics (Table 1).

### 3.2. Oxygen-Ozone Treatment Promotes the Wound Healing of DFUs

**Figure 2:**
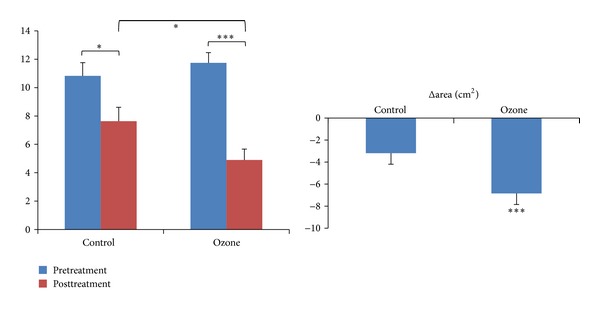
Wound size reduction (cm², $\overset{-}{x}\pm s$). Before treatment there was no significant difference in ulcer area between the ozone group and the control group (11.74 ± 0.72 versus 10.82 ± 0.93, *P* = 0.439). After treatment the ulcer area in both groups was significantly smaller than before. The wound area reduction (Δarea) was significantly more in the ozone group than in the control group (6.84 ± 0.62 versus 3.19 ± 0.65 cm^2^, *P* < 0.001). *P < 0.05, ***P < 0.001.

At day 20 there were 6, 12, 5, and 2 of patients in ozone group that reached grades 3, 2, 1, and 0, respectively, whereas in control group, there were only 3, 7, 6, and 9 of patients that reached grades 3, 2, 1, and 0, respectively. The effective rate was significantly higher in ozone group than in control group (92% (23/25) versus 64% (16/25), *χ* ^2^ = 5.711,  *P* = 0.037). At baseline there was no significant difference in the wound size between two groups (11.74 ± 0.72 versus 10.82 ± 0.93, *P* = 0.439). At day 20 after treatment the wound size in both groups was significantly smaller than before (*P* values were <0.001 and 0.022, resp.). In ozone group the wound size reduction was significantly more than in control group (6.84 ± 0.62 versus 3.19 ± 0.65 cm^2^, *P* < 0.001) (Figure 2).

### 3.3. Oxygen-Ozone Treatment Increased the Collagen Contents of the Wounds

**Figure 3:**
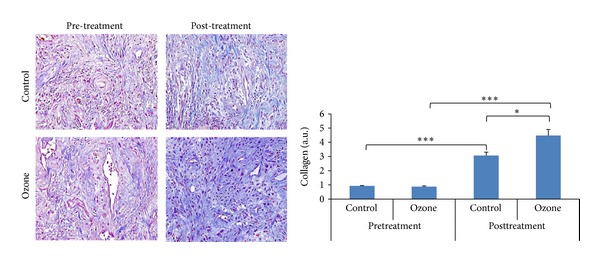
Collagen fibers in tissue specimens by Masson's staining (×40). Before treatment there was no difference in collagen fibers between the ozone group and the control group (0.92 ± 0.04 versus 0.88 ± 0.05, *P* = 0.433). After treatment there were more collagen fibers than before in both groups (*P* < 0.001). The collagen fibers were significantly more in the ozone group than in the control group (4.48 ± 0.43 versus 3.07 ± 0.23, *P* = 0.012). *P < 0.05, ***P < 0.001.

At baseline there was no difference in the quantity of collagen fibers between two groups (0.92 ± 0.04 versus 0.88 ± 0.05, *P* = 0.433). After treatment there were more collagen fibers than before in both groups (*P* < 0.001). But in ozone group the collagen fibers were more than in the control group (4.48 ± 0.43 versus 3.07 ± 0.23, *P* = 0.012) (Figure 3).

### 3.4. VEGF, TGF-*β*, and PDGF Levels in Wound Exudates Were Upregulated by Ozone Therapy

**Figure 4:**
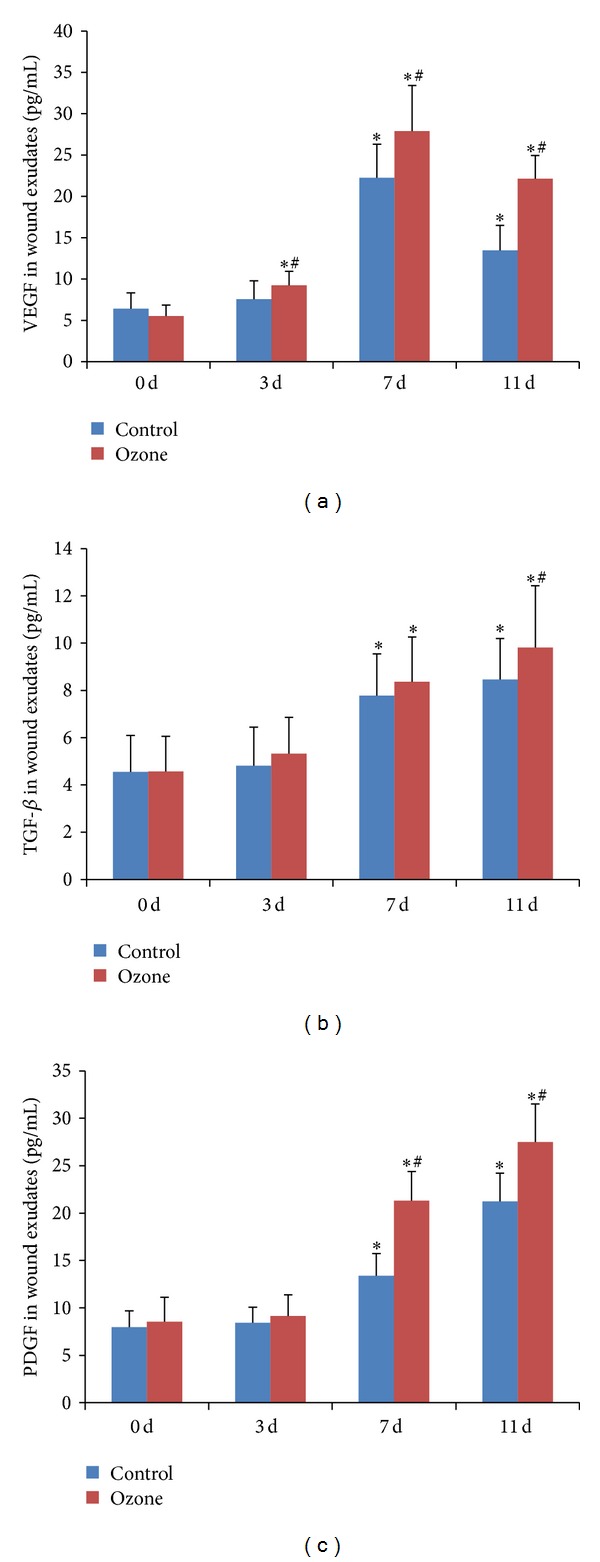
Changes of growth factors in wound exudates at 0, 3, 7, and 11 d after treatment. (a) VEGF levels (pg/mL) in wound exudates significantly increased in both groups after treatment. (b) TGF-*β* levels (pg/mL) in wound exudates significantly increased in both groups at 7 d and 11 d after treatment. And at 11 d ozone group has higher TGF-*β* level than control. (c) PDGF levels (pg/mL) in wound exudates significantly increased at 7 and 11 d after treatment in both groups with higher levels in ozone group than in control. *P < 0.05 versus the same group at day 0. ^#^ *P* < 0.05 versus control group at the same day.

At days 3, 7, and 11 after treatment, VEGF levels in wound exudates significantly increased in both groups. At days 7 and 11 the levels of VEGF and PDGF were significantly higher in ozone group than in control (27.89 ± 5.53 versus 22.25 ± 4.05, *P* < 0.05; 21.31 ± 3.08 versus 13.39 ± 2.33, *P* < 0.05). The levels of TGF-*β* and PDGF were significantly increased in both groups at 7 d and 11 d after treatment (*P* < 0.05). At day 11 the ozone group has significantly higher TGF-*β* level than control (9.81 ± 2.61 versus 8.45 ± 1.74; *P* < 0.05) (Figure 4).

### 3.5. Contents of VEGF, TGF-*β*, and PDGF in Tissues

**Figure 5:**
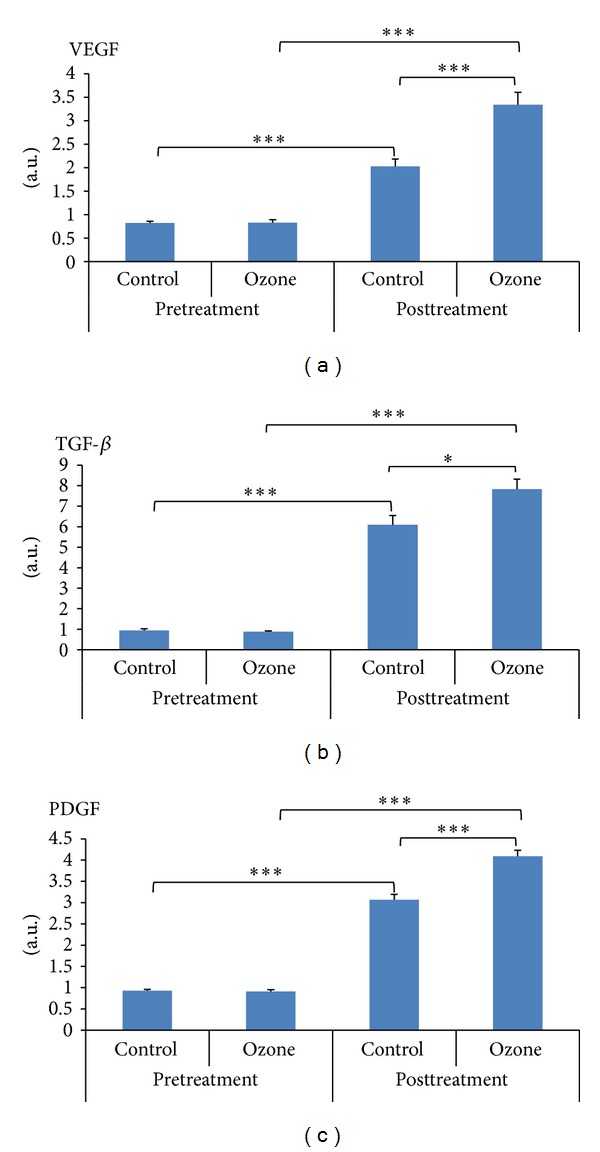
Contents of VEGF, TGF-*β*, and PDGF in tissue specimens (pg/mg). Before treatment there were no significant differences in the contents of VEGF, TGF-*β*, and PDGF in the wound between the ozone group and the control group (19.95 ± 0.53 versus 17.93 ± 0.84, *P* = 0.056; 4.48 ± 0.43 versus 5.17 ± 0.49, *P* = 0.304; 14.23 ± 0.68 versus 15.50 + 0.78, *P* = 0.235). But after treatment the contents of VEGF, TGF-*β*, and PDGF were all significantly higher in the ozone group than in the control group (34.86 ± 3.00 versus 26.44 ± 2.02, *P* = 0.032; 14.95 ± 1.39 versus 10.45 ± 1.07, *P* = 0.019; 31.44 ± 3.33 versus 20.78 ± 2.69, *P* = 0.023). *P < 0.05, ***P < 0.001.

At baseline there were no significant differences in the contents of VEGF, TGF-*β*, and PDGF in the wound between two groups (19.95 ± 0.53 versus 17.93 ± 0.84, *P* = 0.056; 4.48 ± 0.43 versus 5.17 ± 0.49, *P* = 0.304; 14.23 ± 0.68 versus 15.50 ± 0.78, *P* = 0.235). But after treatment at day 11 they were significantly higher in ozone group than in control group (34.86 ± 3.00 versus 26.44 ± 2.02, *P* = 0.032; 14.95 ± 1.39 versus 10.45 ± 1.07, *P* = 0.019; 31.44 ± 3.33 versus 20.78 ± 2.69, *P* = 0.023) (Figure 5).

### 3.6. Expressions of VEGF, TGF-*β*, and PDGF Proteins in Tissues

**Figure 6:**
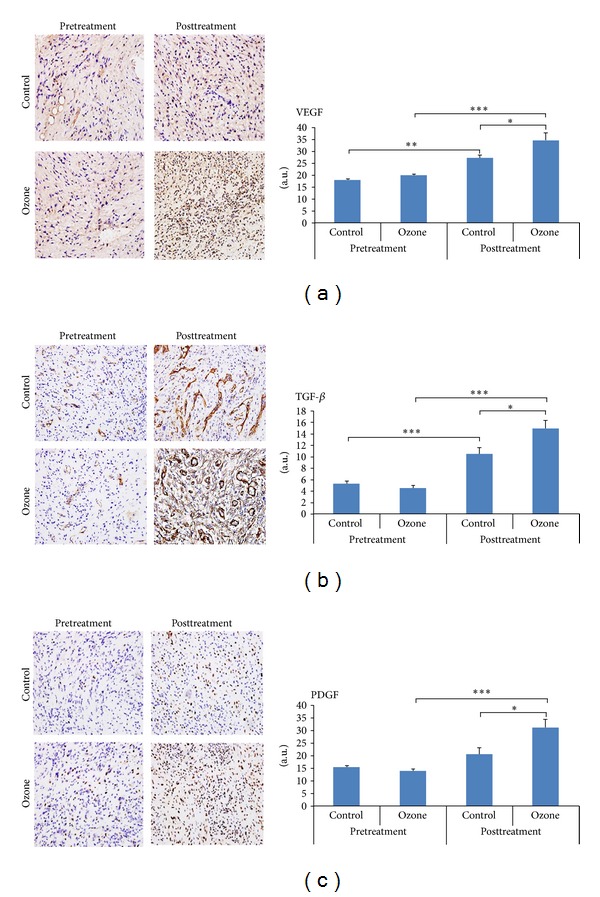
Expressions of VEGF, TGF-*β*, and PDGF by immunohistochemical examinations (×40). (a) Expressions of VEGF pre- and posttreatment in two groups. (b) Expressions of TGF-*β* pre- and posttreatment in two groups. (c) Expressions of PDGF pre- and posttreatment in two groups. Before treatment there were no significant differences in the expressions of VEGF, TGF-*β*, and PDGF protein between the ozone group and the control group (0.83 ± 0.06 versus 0.82 + 0.04, *P* = 0.892; 0.88 ± 0.05 versus 0.94 ± 0.08, *P* = 0.495; 0.91 + 0.04 versus 0.92 ± 0.04, *P* = 0.802). But after treatment the expressions of VEGF, TGF-*β*, and PDGF were all significantly higher in the ozone group than in the control group (3.34 ± 0.27 versus 2.03 ± 0.16, *P* < 0.001; 7.83 ± 0.49 versus 6.10 ± 0.45, *P* = 0.018; 4.09 ± 0.14 versus 3.06 ± 0.13, *P* < 0.001). *P < 0.05, ***P < 0.001.

At baseline there were no significant difference in the expressions of VEGF, TGF-*β*, and PDGF protein between two groups (0.83 ± 0.06 versus 0.82 ± 0.04, *P* = 0.892; 0.88 ± 0.05 versus 0.94 ± 0.08, *P* = 0.495; 0.91 ± 0.04 versus 0.92 ± 0.04, *P* = 0.802). But after treatment at day 11 they were significantly higher in ozone group than in control group (3.34 ± 0.27 versus 2.03 ± 0.16, *P* < 0.001; 7.83 ± 0.49 versus 6.10 ± 0.45, *P* = 0.018; 4.09 ± 0.14 versus 3.06 ± 0.13, *P* < 0.001) (Figure 6).

## 4. Discussions

DFU is a major cause of amputation, which is caused by both vascular and neurologic complications of diabetes, in combination with persistent opportunistic infections and deficient wound healing. The management of DFUs is a great challenge for clinicians. The ozone treatment (1.1 mg/kg with an ozone concentration of 50 *μ*g/mL via rectal insufflation) can improve glycemic control and prevent oxidative stress in STZ-induced diabetic rats [11, 12]. Most recently, ozone administered was reported to prevent atherosclerosis development and increase antioxidant systems in New Zealand white rabbits [13]. The antioxidative effects of ozone also ameliorate the age-related biochemical changes in male rat cerebral cortex [14]. In patients with coronary artery disease (CAD), ozone treatment by rectal insufflation significantly improved prothrombin time, reduced biomarkers of protein and lipid oxidation, and increased total antioxidant [15]. A randomized controlled clinical trial in diabetic patients with peripheral arterial diseases (PAD) and diabetic foot showed improved glycemic control, reduced the area of lesions, inhibited oxidative stress and fewer amputations in patients treated with ozone for 20 days via rectal insufflation than in the control group [16]. Wainstein et al. reported that the ozone treatment in addition to the conventional treatment for 24 weeks was superior to conventional treatment alone in promoting the complete healing of DFUs [10].

DFU is characterized by impaired wound healing, delayed closure time, and decreased collagen deposition associated with reduced expressions of endogenous growth factors in the wound [17]. Multiple growth factors, such as VEGF, TGF-*β*, and PDGF, play an important role in wound healing. Lack of upregulation of some angiogenic and leukocyte chemotactic factors may account for a poor formation of granulation tissue and chronicity of ulcer epithelialization [18]. Treatments increasing the expressions or levels of growth factors have been proven to be effective in the wound healing of DFUs [19]. The scaffold containing recombinant human VEGF and basic fibroblast growth factor (bFGF) significantly accelerated wound closure at day 15 compared to scaffolds without growth factors in db/db mice [20]. However, the local application of growth factors has shown poor efficiency due to the rapid leakage and short half-life of growth factors at the wound bed. Thus, it may be more important for the wound healing to stimulate the expressions of endogenous growth factors at local wound site.

In our study, the oxygen-ozone treatment significantly promoted the early effective rate of the wound healing at day 20 in DFU patients. We also observed that there were significantly higher expressions of VEGF, TGF-*β*, and PDGF in the ozone group than in the control group. The results show that the efficacy of the ozone treatment for the healing of DFUs may be partially due to the increasing of endogenous growth factors in the local wounds, which has not been reported before.

## Figures and Tables

**Table 1:** General data at baseline between ozone group and control group.

	Control	Ozone	*t*/*χ* ^2^/*z*-value	*P* value
	*n* = 25	*n* = 25		
Age (yrs)	61.12 ± 10.90	59.72 ± 12.20	0.428	0.671
Gender (male/female)	12/13	14/11	0.571	0.778
Duration of DM (yrs)	10.24 ± 5.47	8.64 ± 5.35	1.045	0.301
Duration of DFU (days)	46.60 ± 10.79	45.04 ± 8.59	0.113	0.910
SBP (mmHg)	136.96 ± 19.06	142.36 ± 23.61	0.890	0.378
DBP (mmHg)	77.80 ± 7.50	81.88 ± 13.50	1.321	0.193
HbA1c (%)	8.43 ± 1.78	8.56 ± 1.75	0.924	0.793
Hb (g/L)	113.92 ± 16.01	114.24 ± 20.24	0.062	0.951
CR (umol/L)	70.80 ± 28.99	80.72 ± 34.95	1.092	0.280
AST (U/L)	19.41 ± 5.27	20.51 ± 7.47	0.600	0.552
ALT (U/L)	15.10 ± 6.19	18.11 ± 8.33	1.451	0.153
LDL (mmol/L)	2.59 ± 0.75	2.73 ± 0.89	2.733	0.887
ABI	0.97 ± 0.18	1.00 ± 0.23	0.620	0.538
Wagner stage 2/3/4	13/9/3	11/10/4	0.596	0.511
